# Effects of Whole-Body Vibration and Manually Assisted Locomotor Therapy on Neurotrophin-3 Expression and Microglia/Macrophage Mobilization Following Thoracic Spinal Cord Injury in Rats

**DOI:** 10.3390/cimb45040211

**Published:** 2023-04-07

**Authors:** Diana Schaufler, Maria Eleni Manthou, Paschalis Theotokis, Svenja Rink-Notzon, Doychin N. Angelov

**Affiliations:** 1 Department I of Internal Medicine, Lung Cancer Group Cologne, University Hospital Cologne, 50931 Cologne, Germany; 2 Anatomical Institute II, University of Cologne, 50931 Cologne, Germany; 3 Department of Histology and Embryology, Aristotle University Thessaloniki, 54124 Thessaloniki, Greece; 4 Laboratory of Experimental Neurology and Neuroimmunology, Second Department of Neurology, AHEPA University Hospital, 54124 Thessaloniki, Greece; 5 Department of Prosthetic Dentistry, School of Dental and Oral Medicine, University of Cologne, 50931 Cologne, Germany

**Keywords:** microglia, Iba1, neurotrophin-3, spinal cord injury, vibration therapy, functional recovery

## Abstract

Microglial cells play an important role in neuroinflammation and secondary damages after spinal cord injury (SCI). Progressive microglia/macrophage inflammation along the entire spinal axis follows SCI, and various factors may determine the microglial activation profile. Neurotrophin-3 (NT-3) is known to control the survival of neurons, the function of synapses, and the release of neurotransmitters, while also stimulating axon plasticity and growth. We examined the effects of whole-body vibration (WBV) and forms of assisted locomotor therapy, such as passive flexion–extension (PFE) therapy, at the neuronal level after SCI, with a focus on changes in NT-3 expression and on microglia/macrophage reaction, as they play a major role in the reconstitution of CNS integrity after injury and they may critically account for the observed structural and functional benefits of physical therapy. More specifically, the WBV therapy resulted in the best overall functional recovery when initiated at day 14, while inducing a decrease in Iba1 and the highest increase in NT-3. Therefore, the WBV therapy at the 14th day appeared to be superior to the PFE therapy in terms of recovery. Functional deficits and subsequent rehabilitation depend heavily upon the inflammatory processes occurring caudally to the injury site; thus, we propose that increased expression of NT-3, especially in the dorsal horn, could potentially be the mediator of this favorable outcome.

## 1. Introduction

Whole-body vibration (WBV) and assisted locomotor therapy are used in the activity-based clinical rehabilitation of patients with neurological impairments, such as spinal cord injury (SCI). The application of these therapies in neurorehabilitation is primarily due to their functional benefits, although a complete understanding the concomitant neuronal effects on the spinal cord or brain is lacking [1]. The physiological processes behind the therapeutic benefits are based on a variety of hypotheses [2]. However, currently, the inability to fully comprehend them stands as an impediment to offering scientifically based, optimal guidelines for treatment [1].

The pathophysiology of SCI is very complicated; nevertheless, it is well known that microglia play an important role in neuroinflammation and secondary damages arising from SCI [3,4]. Microglial cells constitute the resident tissue macrophages in the CNS and constantly search for changes caused by injuries [3,5]. During neuroinflammation, microglia, which are known as a double-edged sword in SCI, can aid in the healing of the injured spinal cord, but they can also have a harmful effect by secreting an excessive amount of cytotoxic cytokines and reactive oxygen mediators [6]. Tissue recovery and protective effects can be mediated by lesion containment, debris clearance, and the production of anti-inflammatory factors [4,6]. However, they can also be implicated in nerve degeneration [7,8] and might have negative effects on myelin and residual oligodendroglia [9].

Following SCI, a progressive neurodegeneration and demyelination of lesioned axons occurs in both gray and white matter, in what is termed Wallerian degeneration [10,11], which coincides with the activation of microglia/macrophages [10,11,12]. Studies demonstrated that progressive microglia/macrophage inflammation along the entire spinal axis follows thoracic SCI [13,14,15]. Various factors may determine the microglial activation profile and different microglial activation profiles have been described in studies [16,17,18]; however, accurate time-course profiles of microglial activation have not yet been demonstrated in various SCI models [3].

Neurotrophin-3 (NT-3) belongs to a family of proteins that control the survival of neurons, proper functionality at the synapse level, and the release of neurotransmitters, while also stimulating axon plasticity and growth [19]. They were shown to have beneficial effects on the development, survival, and direction of damaged neurons in the spinal cord, which makes them promising alternatives for use in treatment plans after SCI [19].

It has been implied that neuronal and synaptic activity modulate NT-3 expression and microglia/macrophage function in the CNS [20,21,22]. In this study, we investigated the impact of physical therapy on neuroimmune interactions and functional recovery after thoracic compressive SCI. The aim was to examine the effects of WBV and passive flexion-extension (PFE) therapy at the neuronal level after SCI, with a focus on changes in NT-3 expression and on microglia/macrophage reactions, as they play a major role in the reconstitution of CNS integrity after injury and they may critically account for the observed structural and functional benefits of physical therapy.

## 2. Materials and Methods

### 2.1. Animal Groups

We based our study on previously published data [23,24] about the effects of WBV on rats with SCI. Following severe compressive SCI at a low thoracic level (Th8), rats were subjected to WBV starting at either day 7 (WBV7 group, *n* = 10), day 14 (WBV14 group, *n* = 10), or day 28 post-injury (WBV28 group, *n* = 10). Some of the animals were designated to receive manually assisted locomotor therapy, particularly passive flexion-extension (group PFE, *n* = 10), which started at day 14 post-injury. Rats subjected to SCI with no further therapeutic intervention (SCI-noEx group, *n* = 10) served as controls. Intact rats (*n* = 3) set a baseline value for structural and functional parameters (Table 1). There were no unexpected fatalities in any of the groups of rats, which were evaluated at evenly spaced periods of 1, 3, 6, 9, and 12 weeks following SCI.

### 2.2. Pre-Injury Conditioning

A total of 53 adult female Wistar rats (strain HsdCpb:WU, Harlan-Winkelmann, Borchen, Germany, 3–6 months of age, 175–200 g in weight) were used in this study. Animals were housed at 23 °C with a 12-h artificial light–dark cycle and fed standard laboratory food (Ssniff, Soest, Germany). Tap water was also made available to them as needed. The local Animal Care Committee approved all methods and tests in conformity with German Law on the Protection of Animals (approval number 8.87-50.10.35.08.144).

All 53 rats underwent a pre-injury (pre-operative) conditioning phase of 2 weeks with daily exercise of beam walking and inclined ladder climbing to adjust to subsequent locomotor tests in the post-injury (post-operative) trial phase. In a rat Galileo chamber, the animals that were chosen to receive WBV were adapted to it for 2 min at 15 Hz and 1 min at 30 Hz, five days a week, for two weeks. The PFE was administered to animals who were chosen to receive it following SCI for two weeks. Within two to three days, all animals quickly adjusted to WBV and PFE conditioning and exhibited no indicators of stress, such as freezing or attempting to bite, weight loss, or lack of grooming.

### 2.3. Spinal Cord Injury

**Figure 1:**
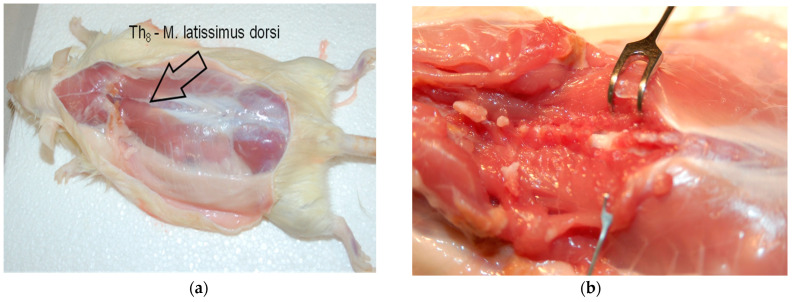
(**a**) The upper margin of the latissimus dorsi muscle (arrow) marks the laminectomy and injury level at Th8; (**b**) the prominent processus spinosus of the second thoracic vertebra was used as a landmark. Laminectomy and injury level at Th8.

**Figure 2:**
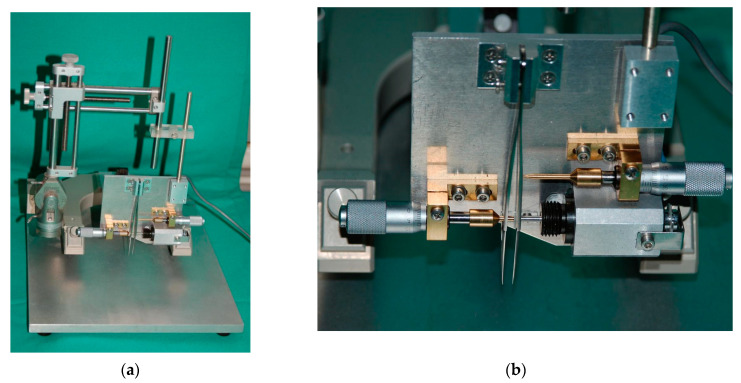
(**a**) The custom-made spinal-cord-compression device (Curtis et al.,1993) [26] was attached to a stereotaxic frame and mounted on a metal block; (**b**) controlled by an electromagnetic drive and timed current, the drive pin closes the forceps by pressing the moving blade. The degree of closure of the forceps can be adjusted by the limiting pin, which passes through a drilled hole in the stationary blade. The closing pin is used to advance the moving blade and thus position the tips of the forceps accurately next to the spinal cord. The use of calibrating screws allows vernier adjustment and, thus, the measurement and standardization of the SCI (Apostolova et al., 2006; Manthou et al., 2015; Semler et al., 2011) [25,27,28].

Severe spinal cord compression was performed according to a method we described in a previously published work [23,24,25]. Briefly, laminectomy at Th8 level was conducted on rats under general anesthesia (1.8 vol.% isofluorane: Forene, Abbott, Germany, 0.6 l/min O_2_: Conoxia, Linde, Germany and 1.2 l/min N_2_O: Niontix, Linde, Germany) (Figure 1). The exposed 10th thoracic section of the spinal cord was compressed with electromagnetically controlled watchmaker forceps (Dumont #5, Fine Science Tool, Heidelberg, Germany). The forceps’ closure was set at 50% of spinal cord diameter (Figure 2). The Basso, Beattie, and Bresnahan (BBB) score after the contusion was between 1 and 2 within 1 week of injury. The rats were kept at 37 °C for 12 h to avoid hypothermia prior to being separated in conventional cages. Up until the experiment’s conclusion (12 weeks after SCI) their bladders were manually emptied three times per day.

### 2.4. Whole-Body Vibration (WBV) Therapy

**Figure 3:**
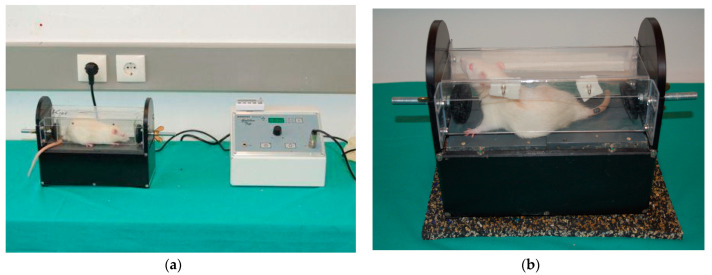
(**a**,**b**) Whole-body vibration (WBV) therapy. The WBV therapy was applied by the custom-made Galileo-system device. Pre-injury conditioning started 2 weeks prior to SCI and post-injury treatment started on day 7, 14, or 28 after SCI. Every WBV session comprised five sequential trials, each consisting of 1-min 15-Hz vibration followed by 2 min at 30 Hz [28].

Whole-body vibration was delivered utilizing a specially constructed Galileo system device (Novotec Medical GmbH, Pforzheim, Germany), as documented in our previous work [23,29]. The chamber’s design allowed animals to enter voluntarily and to move and turn around, relieving stress. Two plates that made up the chamber floor independently vibrated in opposite directions, alternately moving up and down (1.5 mm amplitude; 3 mm peak-to-peak distance). We chose one amplitude (1.5 mm) and two electronically regulated frequencies (15 Hz and 30 Hz), which were tolerated well by the rats, fell within the usual range of motoneuron discharges, and did not exceed forces during typical weight-bearing locomotion. Rats were exposed to WBV five days a week, once every day. Each WBV session consisted of five consecutive trials, each of which featured one minute at 15 Hz followed by two minutes at 30 Hz, for a total of three minutes. A one-minute rest was allowed between each unit to prevent tiredness and muscle fatigue. Each rat’s entire body experienced alternating vibrations that resembled rocking, with an amplitude of up to 1.5 mm and a frequency of 15 or 30 Hz, as a result of the motions of the floor plates (Figure 3).

### 2.5. Passive Flexion–Extension (PFE)

**Figure 4:**
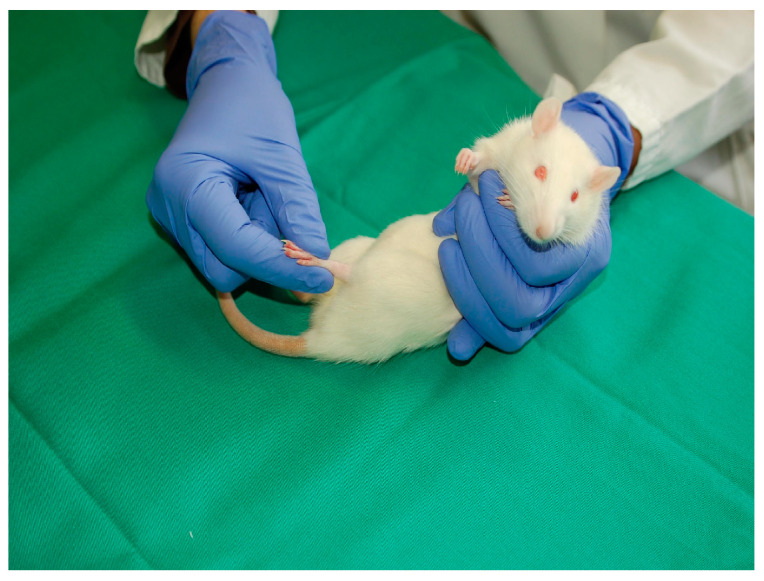
Manually assisted locomotor (ML) therapy, passive flexion–extension (PFE) exercise. The conditioning started 2 weeks prior to SCI and post-injury treatment started on day 14 after SCI. Each ML (PFE) treatment session consisted of a total of 80 alternating passive flexions and extensions in all three joints of the hindlimbs (i.e., hip, knee, and ankle joints).

To test whether frequent, passive stretching of the paralyzed intrafusal muscle spindles might be beneficial after SCI, the PFE group was utilized as a control group. Before each treatment session, the animals inhaled a short-duration anesthetic to facilitate manual handling and to prevent unnecessary stress on the animals. Beginning on the 14th day after operation, treatment was performed holding the animals, which lay on their backs, in one palm of the hand with all four limbs kept off the ground (no limb-loading) (Figure 4). Hindlimbs were further passively moved at all three joints, i.e., hip, knee, and ankle joints, by a total of 80 alternating flexions and extensions, for 1 min twice a day. Through type Ia and type II fibers during extension, the method moves joints in a manner that mimics gait [30,31,32] and offers trophic support to spinal motoneurons [33,34,35].

### 2.6. Locomotor Performance

Utilizing the BBB rating scale, locomotor function was assessed [36]. Two independent researchers blinded to treatment performed the scoring. Using a walking platform (referred to as a “beam”), locomotor function was also evaluated, as previously described [23,25]. Video recordings (Panasonic NVDS12 at 25 frames per sec) were made after pre-conditioning training, just before SCI and throughout testing at 1, 3, 6, 9, and 12 weeks after SCI. Animals were not exposed to beam walking between recording sessions. Using ImageTool 2.0 software, selected frames were utilized to calculate the rump-height index (RHI) and footstepping angle (FSA) [23,27]. Analyses were performed by two investigators blinded to the treatment.

### 2.7. Tissue Preparation

Twelve weeks after SCI marked the endpoint of the experiment and animals were sacrificed. The vertebral canal was dissected and a bilateral single-level laminectomy of the second lumbar vertebra was performed to expose the spinal L2 region. A 3-mm-thick segment from the widest part of the lumbar enlargement was taken. Following cryoprotection in 20% sucrose in PBS overnight, serial transverse sections, 25 μm thick, were obtained on a cryostat (CM3050; Leica, Nussloch, Germany).

### 2.8. Immunohistochemistry

An average of 10 sections at 250 µm rostral-to-caudal intervals (i.e., every 10th section) were mounted on SuperFrost Plus slides (Roth, Karlsruhe, Germany), so that each slide had 10 sections representative of the entire lumbar-spinal-cord segment [27]. Sodium-citrate solution (0.01M, pH 9.0) was used for water-bath antigen retrieval for 30 min at 80 °C. Sections were washed for 5 min at room temperature in PBS (0.1M, pH 7.4). Normal sheep serum (NSS), diluted in PBS (0.1 M, pH 7.4), containing 0.2 percent *v*/*v* Triton X-100 (facilitating antibody penetration), and 0.02 percent *w*/*v* sodium azide (which prevents microbiological contamination), was used to block non-specific binding for two hours at room temperature. 

Rabbit polyclonal anti-Iba1 antibody (Synaptic Systems, Göttingen, Germany, Cat. Nr. 234003) raised against ionized calcium binding adapter molecule 1 (Iba1), a microglia/macrophage-cell-surface molecule, was used as primary antibody to identify both resting and activated microglia and blood-derived infiltrating macrophages. The antibody Iba1 is acknowledged as a specific identity and activation marker for microglia/macrophages and their associated neuroinflammatory responses [13,37,38,39,40]. Incubation with the anti-Iba1 primary antibody (SySy, 234003) diluted 1:1000 in PBS (0.1 M, pH 7.4) and supplemented with 0.5% *w*/*v* λ-carrageenan (Sigma) and 0.02 % *w*/*v* sodium azide, was performed for 72h at 4 °C; λ-carrageenan is a non-gelling vegetable gelatin, which reduces nonspecific binding and stabilizes the antibody solution. Neuronal cell bodies were stained using NeuroTrace 500/525 fluorescent green Nissl solution (1:200, molecular probes, N21480, from Life Technologies GmbH, Darmstadt, Germany).

Neurotrophin-3 (NT-3) immunostaining was performed similarly, as described above. The primary antibody used was rabbit polyclonal anti-NT-3 antibody (N-20: sc-546, Santa Cruz Biotechnology), diluted 1:40 in PBS (0.1 M, pH 7.4) and supplemented with 0.5% *w*/*v* λ-carrageenan (Sigma) and 0.02 % *w*/*v* sodium azide.

Immunostained sections were observed under a Zeiss fluorescence microscope Model 1.0.0 S/N 971,006 and imaged at 10× magnification with 3-s exposure time using SpotCam version 3.5 camera system and Visitron System software image tool (Puchheim, Germany). Each acquired image was further digitized and processed using the Image-Pro Plus software version 6.0 (Media Cybernetics, Inc., Silver Spring, MD, USA). The spinal cord sections that were ruined during the process or that had excessive artefacts were excluded from the study. The animals from which we failed to collect at least 9 readings, to obtain a reliable mean value, were also excluded from the study. Fluorescent images were encoded and compared as described by Manthou et al. [23]

### 2.9. Statistical Analyses

Statistical tests were performed using the Sigma Plot 11 software (Chicago, IL, USA) and SPSS (version 18 for Windows, Chicago, IL, USA, SPSS Inc.). A one-way analysis of variance (ANOVA) followed by the Holm–Sidak post hoc test were used to assess significant differences across groups for all data, unless stated otherwise. Kruskal–Wallis one-way ANOVA on ranks followed by Dunn’s post hoc test were performed if the sample variables did not fit a normal distribution or were not equally variant. Spearman’s correlation was used to determine the relationships between Iba1 expression and open-field BBB locomotor performance. Spearman’s correlation coefficient r values were interpreted as follows: 0–0.25 = no or little relationship; 0.25–0.5 = fair relationship; 0.5–0.75 = moderate relationship; 0.75–1.0 = good-to-excellent relationship. Positive r values suggested a positive correlation, whereas negative r values described an inverse correlation. All data are presented as group mean ± standard deviation (SD), unless stated otherwise. For all statistical tests, the significance level was set to *p* < 0.05.

## 3. Results

### 3.1. Effects of WBV and PFE Therapy on Functional Recovery after SCI

The open-field locomotor recovery, as assessed by the BBB rating, and the stepping ability, as determined by the foot stepping angle (FSA) recovery, were not significantly improved by WBV and PFE therapy. A marked trend for improved recovery in both tests was detected in the rats in the WBV14 group. Surprisingly, the FSA scores of the WBV7 and WBV28 groups were inferior to the scores for spontaneous recovery in the SCI-noEx group, indicating that these therapeutic programs compromised functional recovery (Table 2).

During the beam walking, the hindlimbs’ capacity to support the body weight was evaluated by the rump-height index (RHI). At 12 weeks, the WBV14- and PFE-treated rats were able to raise their trunks to roughly 75% of their normal rump height, on average (*p* < 0.05). The correct ladder-step performance was not significantly improved by any of the types of therapy. The statistical tests of the performance revealed no significant differences between the groups.

### 3.2. Immunohistochemical Analysis of Iba1 Expression

The groups’ mean values of Iba1 expression, as defined by the absolute pixel number (PN) in the ventral horn and dorsal horn of the lumbar spinal cord L2 segment of the intact rats and of the injured, treated, and non-treated rats at 12 weeks after SCI, are shown in Table 3.

**Figure 5:**
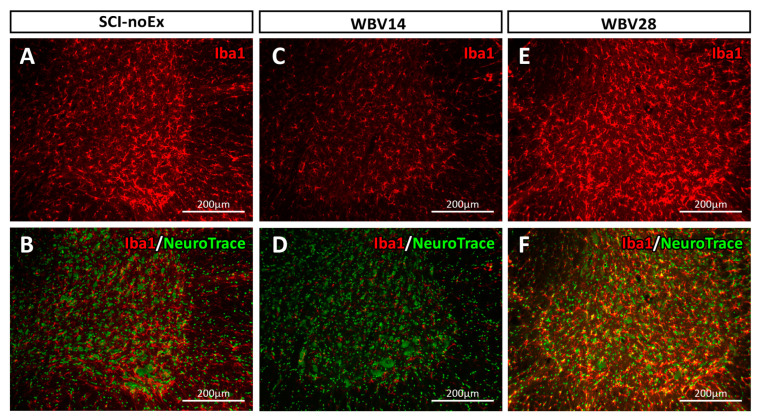
Immunostaining of microglia/macrophages in the ventral horn in a transverse section of the lumbar-spinal-cord L2 segment using rabbit anti-Iba1 and sheep anti-rabbit Cy3 conjugated IgG antibodies at 12 weeks after SCI. The Iba1 immunostaining visualized microglia/macrophages in their characteristic star- or irregular round-shaped cell morphologies with their cell processes, which extended in all directions from the cell body. (**A**) SCI-noEx, (**C**) WBV14, (**E**) WBV28. Counterstaining with NeuroTrace 500/525 green fluorescent Nissl staining unequivocally showed the large neuronal cell bodies of the ventral horn α-motoneurons (**B**) SCI-noEx, (**D**) WBV14, and (**F**) WBV28.

**Figure 6:**
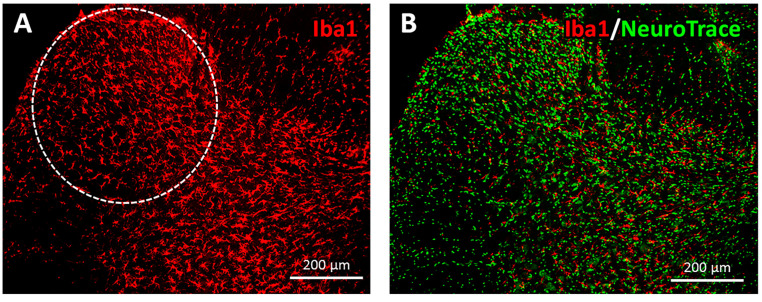
Immunostaining of microglia/macrophages in the dorsal horn in a transverse section of the lumbar-spinal-cord L2 segment using rabbit anti-Iba1 and sheep anti-rabbit Cy3 conjugated IgG antibodies at 12 weeks after SCI (image belongs to WBV28 group). Microglia/macrophages were found predominantly in the gray matter—involving both the ventral horn and dorsal horn—where they were in intimate spatial contact with the neuronal cell bodies (**B**), exposed with green immunofluorescence via counterstaining with NeuroTrace 500/525). The area of interest (AOI) of the dorsal horn is illustrated by a white dashed line (**A**).

The Iba1 expression was predominantly found to be increased in the gray matter, in both the ventral and dorsal horns, with comparatively low expression in the white matter of the lumbar spinal cord. This distribution pattern of the Iba1 was observed in all the experimental groups, i.e., in all the groups with and without SCI or therapy (intact, SCI-noEx, WBV7, WBV14, WBV28, and PFE groups). The Iba1 immunostaining provided a visualization of the microglia/macrophages in their star- and irregular-round-shaped cell morphologies, with their branched cell processes (Figure 5 and Figure 6). The Iba1-positive cells were in intimate spatial contact with α-motoneurons and other interneurons, which were collectively labeled by NeuroTrace green fluorescent Nissl staining (Figure 5 and Figure 6).

The immunohistochemical analysis at 12 weeks after SCI, conducted on all the non-treated rats, revealed a consistent trend of decrease in Iba1 expression (Table 3). However, there were no significant differences in Iba1 expression between the intact and SCI-noEx groups. This was the case for both the ventral and the dorsal horns (*p* > 0.05, Kruskal–Wallis, Dunn’s method, respectively). The correlation analysis showed that the microglia/macrophage inflammatory response in the spinal cords of the SCI-noEx animals was significantly and inversely correlated with functional recovery. The non-exercised animals with higher Iba1 expression, i.e., more intense microglial/macrophage inflammation, exhibited comparatively poor locomotor recovery, and vice versa.

The WBV and PFE therapies showcased an increase in Iba1 expression in both the ventral and dorsal horns of the lumbar spinal cord, after thoracic SCI. This reaction was particularly evident in the WBV7, WBV28, and PFE groups (Table 3). The WBV therapy starting at 28 days post-injury was the only treatment setup to yield significant increase rates in Iba1 expression levels compared to the SCI-noEx groups, particularly in the ventral horn (*p* = 0.028, Kruskal–Wallis, Dunn’s method). Of all the treatment groups, the WBV14 group exhibited the smallest rate of increase in Iba1 expression in both the ventral horn and the dorsal horn, compared to the SCI-noEx group (*p* > 0.05, Kruskal–Wallis, Dunn’s method). A significant difference (*p* = 0.019, Kruskal–Wallis, Dunn’s method) was identified between the two groups, with larger and smaller increases in Iba1 expression (WBV28 vs. WBV14). No significant differences were reported between the expressions of Iba1 in the dorsal horns of all the groups (Table 3).

### 3.3. Immunohistochemical Analysis of NT-3 Expression

The relative NT-3 expression values (PN/mm^2^) were calculated by rectifying the absolute NT-3 expression values or pixel numbers for the specific area of interest (AOI) used in the ventral horn (AOI ≈ 0.5177 mm^2^) and dorsal horn (AOI ≈ 0.2223 mm^2^) in order to allow a direct comparison between the ventral and dorsal horn (Table 4).

**Figure 7:**
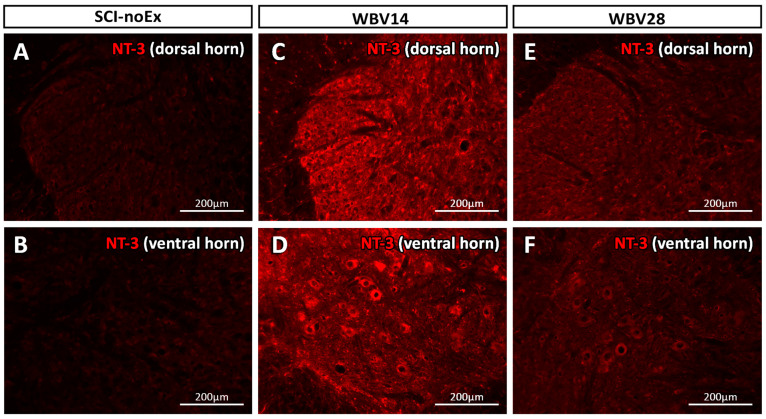
Immunostaining of NT-3 in a transverse section of the lumbar spinal cord L2 segment, using rabbit anti-NT-3 and sheep anti-rabbit Cy3 conjugated IgG antibodies at 12 weeks after SCI NT-3 immunostaining was evident throughout the gray matter, in both ventral horn (**B**,**D**,**F**) and dorsal horn (**A**,**C**,**E**). The NT-3 was highly expressed in α-motoneurons of the ventral horn at WBV14, where we observed clinical recovery. Small cells, presumably interneurons or glial cells, also showed NT-3-positivity. (**A**,**B**) SCI-noEx, (**C**,**D**) WBV14, (**E**,**F**) WBV28.

The immunohistochemical analysis revealed that NT-3 protein expression was evident throughout the gray matter, with only weak staining in the white matter of the lumbar spinal cord. This distribution pattern of NT-3 was observed in all the experimental groups, with and without SCI or therapy. The NT-3 immunostaining in the ventral horn appeared to be located within the α-motoneuron cell bodies (Figure 7). The small cells distributed across the gray matter, presumably interneurons or glial cells, also displayed NT-3-positivity.

The NT-3 expression levels at 12 weeks after SCI were markedly higher in the dorsal horns than in the ventral horns (Table 4). There was also an activity-dependent increase in NT-3 expression in both the ventral and dorsal horns in all the treatment groups (i.e., the WBV7, WBV14, WBV28, and PFE groups). Thus, our results demonstrate that NT-3 increased in all the therapies, independently of the design, task, or time delay in the onset of each therapy, but the increase was only significant in the WBV14 group (*p* < 0.001, WBV14 vs. SCI-noEx, ANOVA, Holm-Sidak).

A correlation analysis revealed a significant and highly positive relationship between the assessed NT-3 expression levels in the ventral horn and the BBB locomotor recovery in the WBV14 group (r = 0.843, *p* = 0.009) at the final assessment time point, 12 weeks post-SCI, which was associated with the overall stronger trend of locomotor recovery from SCI.

## 4. Discussion

Following compressive thoracic SCI, the rats were either subjected to WBV therapy starting at 7, 14, or 28 days post-injury or PFE therapy starting at day 14, or received no exercise. The effects of the selected treatments on functional recovery and on the levels of microglia and neurotrophin-3 varied and, apparently, were dependent on the task and on the time at which each treatment began. The WBV14 therapy appeared to be superior to the PFE therapy in terms of recovery. More specifically, the WBV therapy enhanced the best overall functional recovery when initiated at day 14 and resulted in a moderate increase in Iba1 and the highest increase in NT-3. On the other hand, when the beginning of WBV therapy was either earlier, in a subacute phase 7 days post-injury, or later, on the 28th day, functional recovery was limited, the Iba1 levels were the highest documented, and the NT-3 levels were relatively low.

As verified by the correlation analysis, there was a significant inverse relationship between the Iba1 expression of the ventral horn and the open-field BBB locomotor performance in the non-treated (non-exercised) SCI animals, indicating that microglia/macrophage inflammation impaired neuronal function. The detection of dysfunction or CNS injuries, including trauma, ischemia, infection, neurodegenerative diseases, and even altered neuronal activity, stimulates resting microglia to transform into an activated state. This complex, multistage activation process includes functional and phenotypical changes, such as somatic hypertrophy, thickened branches with microspike formation, and proliferation [7,13,41]. Activated microglia can migrate to the lesion site and release a large number of beneficial and detrimental mediator substances, such as cytokines, chemokines, and neurotrophins, leading to secondary injury and/or repair [7,13,41].

Microglial cells can increase tissue recovery and can exhibit protective effects that are mediated by lesion containment, debris clearance, and the production of anti-inflammatory factors, such as IL-10 [4]. Microglia serve as key cellular components of scars that develop after SCI to protect neural tissue [42]. On the other hand, activated microglia contribute to secondary damage by releasing proinflammatory mediators, such as reactive oxygen species and proinflammatory cytokines [4]. Previous studies found that microglia exhibited at least two different spatial and temporal patterns of activation after compressive SCI. One was rapid, while the other was related to Wallerian degeneration [3]. Further studies demonstrated that progressive microglia/macrophage activation along the entire spinal axis follows thoracic SCI [13,14,15]. This activation was detectable at various timepoints following SCI, immediately, at the subclinical phase, acutely, and chronically, up to at least 6 months later [13]. Although distinct microglial activation profiles have been documented in the literature [16,17,18], and a number of factors may affect them, accurate spatio-temporal profiles of microglial activation have not yet been shown in a variety of SCI models [3].

The SCI-evoked microglia/macrophage inflammatory response in our study was probably composed of the neurotoxic M1 microglia/macrophage subpopulation, which is known to compromise locomotor recovery and to increase secondary tissue damage and neurological dysfunction. It has been suggested that, following SCI, the dynamic lesion environment creates stimuli that control the function of microglia/macrophages and favor the occurrence of this M1 subpopulation, which is considered to impair recovery [43]. This is a “classically-activated”, pro-inflammatory, microglia/macrophage subpopulation, which may be rapidly induced as a robust and chronically persistent response to SCI. This M1 inflammatory response dominates at the injury sites and in the zones of Wallerian degeneration, and propagates mostly neurodestructive reactions [43]. As previously evidenced, the M1 response after SCI is overwhelmingly apparent [43], whereas using WBV later, at day 14, seems to counteract this effect. More importantly, based on the morphologies of the microglial cells in the recovered CNS, microglia/macrophages exhibit the favorable M2 phenotype, which encourages neurorestoration and regeneration [43,44].

The intact blood–spinal-cord barrier (BSB) ensures a stable CNS microenvironment, which is essential for neuronal integrity and function [45,46]. Studies investigating the dynamics of BSB function from several minutes after injury up to 28 days later report time-bound alterations in BSB integrity. Apparently, it opens after injury, followed by a second peak of abnormal permeability 3 days later. It begins to re-establish after 7 days and appears to be largely restored 14 days later. It opens for a second time around the 28th day [45,46,47,48]. This natural behavior of the BSB in the post-SCI period coincides with the time-dependent, phasic response pattern of microglia/macrophages after SCI, as observed by other researchers [13,43,49], and as recorded by their measurements. The rapid and robust microglia/macrophage inflammatory response in the acute post-SCI phase apparently coincides with the first BSB breakdown. On the other hand, the significant reduction in cellular inflammation at the 14 day consistently corresponds with the progressive restoration of the BSB.

Therefore, it appears that microglia/macrophage recruitment and the activation in the spinal cord following SCI are influenced by the integrity of the BSB. The initiation of vibration therapy at the 28th day, when BSB breakdown naturally occurs for the second time in the post-SCI time course, is harmful for the recovery process of the spinal cord and probably exacerbates secondary injuries. The WBV7 intervention did not yield significant changes in Iba1 expression compared to the SCI-noEx group. We can envision that the microglia/macrophages in this early (sub)acute SCI phase become highly sensitized and are therefore more likely to respond quickly and intensively upon their second exposure to pro-inflammatory stimuli. At the 14th day, when BSB integrity is known to be largely re-established, the WBV14 intervention creates a beneficial environment for the spinal cord. This can be accomplished by effectively blocking the further recruitment of immune cells, thus contributing to restored neural function.

With regards to the NT-3, the expression levels at 12 weeks after SCI were markedly higher in the dorsal horns than in the ventral horns. The NT-3 increased with all the therapies, independently of the design, task, or time delay in onset of therapy, although this increase was only significant in the WBV14 group. There was also a significant and highly positive relationship between the assessed NT-3 expression levels in the ventral horn and the BBB locomotor recovery in the WBV14 group, which was associated with a stronger overall trend of locomotor recovery from SCI. In support of this observation, the predominance of NT-3 in the dorsal horns suggests that NT-3 is strongly associated with the sensory neural system and, more specifically, with proprioceptive neurons [50,51]. The NT-3 plays important roles in CNS integrity and function by exerting trophic support for neuronal and glial cells [52,53], by mediating axonal regeneration and the function of sensory neurons [13,43,54,55,56,57], as well as influencing synaptic reformation [58,59,60]. It has been shown to increase in an activity-dependent manner in the intact and injured spinal cords of rats subjected to, for example, treadmill training [61,62], swimming, standing, and stepping training [62,63], bicycle training [63], and wheel running [64].

In animal experiments, NT-3 has been shown to favor axonal regeneration and to improve functional skills [65]. Following trauma-induced denervation, it has been reported, the axonal plasticity of intact CST axons is enhanced by the local, sustained expression of NT-3 [66]. In other studies, NT-3 was shown to stimulate microglial activation and proliferation [52,53]. Coumans et al. [55] used fetal-spinal-cord transplants and neurotrophins (NT-3, BDNF) to provide a permissive environment for axonal regrowth in SCI. They showed that neurotrophins are substantial in neuroregeneration and the increased levels that arise following injury, accompanied by functional recovery, suggests that they are connected to improvement [49,55,56]. Interestingly, increased levels of BDNF, NGF, and NT-3 in the injured spinal cord may be the main therapeutic effect of using hUCB-MSCs to treat spinal cord injuries (SCI) [67].

The absence of off-target effects, such as pain or spasticity, is another benefit of NT-3. In fact, peripheral neuropathies, which are frequently accompanied by persistent pain and allodynia, are currently undergoing treatment with NT-3 in clinical trials. These studies are supported by data showing that NT-3 can enhance functional responsiveness in peripheral sensory neurons and prevent their axons from degenerating [19,68,69,70]. Furthermore, NT-3 has been shown to increase in an activity-dependent manner in the spinal cord following physical exercise. Thus, accumulating data support the concept of a reciprocal neuroimmune interaction between neuronal activity, NT-3, and microglia/macrophages in the CNS. On the other hand, a lack of a profound effect of SCI on NT-3 expression in spinal cord segments caudal to the injury level has also been described in studies [62,71]. Taken together, although NT-3 increased in all the therapies, the increase was only significant in the WBV14 group, positively correlating with functional recovery and inversely correlating with microglia levels.

Another objective of this study was to compare WBV and PFE therapy. Both therapies involved repetitive and rhythmic movements of the impaired lower limbs. However, the modified PFE therapy in our study model did not—in contrast with the WBV therapy—involve the activation of load receptors and plantar cutaneous feedback, which are both acknowledged to exert a strong effect on spinal networks, locomotion capacity, and recovery after SCI [32,72,73,74]. These two sensory inputs are important for the regulation of stance and gait kinematics in healthy and SCI-affected human subjects and in experimental SCI animals [32,72,73,75]. On the other hand, WBV therapy was conducted on non-anesthetized animals, which were free to move on all four paws within the Galileo vibrating device. Providing oscillating stimulation with enhanced reflex and muscle activation [76,77,78,79,80] and, furthermore, the opportunity of free movement within the Galileo device, the WBV therapy was deliberately designed to encourage additional voluntary motor activity. Thus, the WBV therapy incorporated more complex physical training and facilitated the active involvement of the SCI rats.

By design, the WBV therapy combined active and passive (reflex) components of physical activity. The combination of multisensory cues, including load- and hip-joint-related proprioceptive input and plantar cutaneous feedback, appears to be of crucial importance for the generation and regulation of locomotor patterns and the effectiveness of neurorehabilitative physical therapy [32,72,73,75,81,82]. In line with these observations, yet without statistical significance, the complex WBV14 therapy with multi-sensory input led to more improvements overall in over-ground locomotion capacity, left–right-hindlimb coordination, and plantar-stepping performance than the PFE therapy when starting at the same timepoint, on the 14th day post-injury. Surprisingly, even though the PFE therapy did not incorporate limb loading or plantar cutaneous feedback, there was a similar, significant recovery of body-weight support ability and an improved correct ladder-step performance in both the PFE and the WBV14 group.

## 5. Conclusions

Overall, our study used PFE therapy and WBV treatments beginning at various time points in a rat model of severe-compression SCI in order to investigate the correlation between microglia/macrophage inflammation, synaptic restoration, changes in NT-3 expression, and locomotor recovery. Based on our results, the performance and timing of the treatment initiation appear to have a significant impact on the recovery process, since the WBV therapy, starting earlier, at day 14, seemed to be superior to the PFE therapy at the 14th day. The modulatory effects that led to the best overall functional recovery 14 days after SCI included a moderate decrease in Iba1 and a high expression of NT-3 within the dorsal horns of the spinal cord. Evidently, functional recuperation is strongly influenced by the inflammatory processes that take place caudal to the site of damage, and increased NT-3 seems to be the mediator of this recovery.

## Figures and Tables

**Table 1:** Schematic representation of the timeline and the protocols followed.

Group	Day 0	Day 7	Day 14	Day 28	Day 90
Intact					endpoint
SCI					endpoint
SCI		WBV			endpoint
SCI			WBV		endpoint
SCI				WBV	endpoint
SCI			PFE		endpoint

**Table 2:** Time course of the changes in stepping ability of the experimental groups, as assessed by the foot-stepping angle (FSA). Group’s mean values ± SD are shown.

Group	0 Weeks after SCI	1 Week after SCI	3 Weeks after SCI	6 Weeks after SCI	9 Weeks after SCI	12 Weeks after SCI
SCI-noEx	18 ± 1.0	130 ± 20	97 ± 47	88 ± 41	72 ± 41	66 ± 42
WBV7	19 ± 1.0	123 ± 22	103 ± 43	93 ± 47	85 ± 40	93 ± 35
WBV14	18 ± 1.0	98 ± 27	28 ± 21	28 ± 24	26 ± 11	26 ± 11
WBV28	17 ± 1.6	114 ± 49	111 ± 24	86 ± 38	103 ± 39	118 ± 29
ML (PFE)	16 ± 1.0	145 ± 6	132 ± 12	106± 27	103 ± 22	105 ± 36

**Table 3:** Iba1 expression, as defined by absolute pixel number (PN) in the ventral horns and dorsal horns of the lumbar-spinal-cord L2 segments of intact rats and injured, treated, and non-treated rats at 12 weeks after SCI. Groups’ mean values ± SD are shown. The WBV28 therapy led to a significant increase in Iba1 expression in the ventral horn (asterisk; *p* = 0.028, WBV28 vs. SCI-noEx; *p* = 0.019, WBV28 vs. WBV14; Kruskal–Wallis, Dunn’s method).

Group	Iba1_Ventral Horn	Iba1_Dorsal Horn
Intact (*n* = 3)	12,263 ± 1530	13,194 ± 3113
SCI-noEx (*n* = 5)	4535 ± 4234	3868 ±2959
WBV7 (*n* = 5)	12,211 ± 4467	5593 ± 2541
WBV14 (*n* = 9)	6477 ± 2800	5317 ± 2739
WBV28 (*n* = 6)	22,645 ± 11,121 *	12,924 ± 5657
ML (PFE) (*n* = 7)	12,229 ± 6923	7593 ± 4133

**Table 4:** NT-3 expression, as defined by pixel number per mm^2^ of tissue area (PN/mm^2^) in the ventral horns and dorsal horns of the lumbar-spinal-cord L2 segments of intact and injured, treated, and non-treated rats at 12 weeks after SCI. The relative NT-3 expression values (PN/mm^2^) were calculated by rectifying the absolute NT-3 expression values or pixel numbers for the specific AOI used in the ventral horn (AOI ≈ 0.5177 mm^2^) and dorsal horn (AOI ≈ 0.2223 mm^2^) in order to allow direct comparison between the ventral horn and dorsal horn. Groups’ mean values ± SD are shown. Asterisks (*) mark significant results (*p* < 0.001, WBV14 vs. SCI-noEx, ANOVA, Holm-Sidak).

Group	NT-3_Ventral Horn	NT-3_Dorsal Horn
Intact (*n* = 3)	162,483± 9224	481,178 ± 27,187
SCI-noEx (*n* = 5)	176,548 ± 26,117	505,795 ± 53,792
WBV7 (*n* = 5)	334,924 ± 51,345	595,022 ± 61,196
WBV14 (*n* = 9)	525,418 ± 197885 *	739,444 ± 90,572 *
WBV28 (*n* = 6)	318,238 ± 61485	644,613 ± 95,059
ML (PFE) (*n* = 7)	388,171 ± 141,477	624,435 ± 97,267

## Data Availability

The data that support the findings of this study are available from the corresponding author, upon reasonable request.
